# Concomitants of Order Statistics from a Bivariate Generalized Linear Exponential Distribution: Theory and Practice

**DOI:** 10.3390/e28010018

**Published:** 2025-12-24

**Authors:** Areej M. AL-Zaydi

**Affiliations:** Department of Mathematics and Statistics, Faculty of Science, Taif University, P.O. Box 11099, Taif 21944, Saudi Arabia; areej.f@tu.edu.sa

**Keywords:** best linear unbiased estimator, bivariate generalized linear exponential distribution, concomitants of order statistics, ranked set sampling

## Abstract

This paper investigates the concomitants of order statistics from the bivariate generalized linear exponential (BGLE) distribution. We obtain the probability density function of a single concomitant and the joint probability density function of two concomitants of order statistics from the BGLE distribution. In addition, expressions for the single and product moments of concomitants of order statistics are derived. Furthermore, we find the best linear unbiased estimator of a scale parameter related to a study variable using various ranked set sampling techniques. Finally, we apply the findings to a real-life dataset.

## 1. Introduction

The linear exponential (LE) distribution has applications in various fields, including applied statistics and reliability analysis. The LE distribution is also known as the linear failure rate (LFR) distribution. The LE distribution is quite useful in modeling lifetime data with a linear increasing failure rate function, and it includes the exponential and Rayleigh distributions as submodels. In the literature, numerous generalizations of the LE distribution have been presented and investigated. Ref. [1] proposed the generalized linear failure rate (GLFR) distribution. The hazard rate function (HRF) of the GLFR distribution can take various shapes, making it adaptable and suitable for a wide range of survival data sets. The GLFR distribution generalizes numerous well-known distributions, including LFR, generalized exponential, and generalized Rayleigh distributions. Another generalization of the LE distribution was suggested by [2], which is known as the generalized linear exponential (GLE) distribution. Recently, Ref. [3] presented the exponentiated generalized linear exponential (EGLE) distribution, which generalizes the GLFR and GLE distributions.

Bivariate lifetime data is frequently encountered in many practical scientific scenarios. Therefore, it is critical to consider various bivariate models that could be utilized to model such bivariate lifetime data. These models are of interest in a variety of applications, including computer systems, reliability engineering, and Olympic games. The literature has many proposals and investigations into bivariate exponential distributions and their extensions; see, for instance, [4,5,6,7,8,9,10,11,12]. In 2022, Pathak and Vellaisamy introduced a novel family of bivariate generalized linear exponential (BGLE) distributions whose univariate marginals are members of the GLE distribution. They also investigated its various statistical properties. Owing to the presence of five parameters, the joint probability density function (PDF) of the BGLE distribution is very flexible and can take on various shapes depending on the values of the parameters. The BGLE distribution has a bivariate generalized exponential (BGE) distribution and a bivariate generalized Rayleigh (BGR) distribution as sub-models for particular values of parameters. The joint cumulative distribution function (CDF), joint PDF, and conditional PDF for the BGLE distribution are all in closed forms, making them suitable for practical usage. Furthermore, they can be used to model bivariate lifetime data in a variety of situations.

Concomitant or induced order statistics (OSs) were first introduced in the early 1970s by [13,14]. In brief, if there is a sample from a bivariate distribution arranged by the first variable, the second variable associated with the $i^{th}$ first variable is called the concomitant of the $i^{th}$ OS. To review the fundamental results on concomitants of order statistics (COSs), refer to [15]. COSs have found numerous applications in the areas of selection procedures, engineering, inference and prediction issues, and double sampling plans. For a brief overview of COS applications, refer to [16] and the references therein. Several authors have investigated the COSs, including [17,18,19,20,21,22,23,24,25].

Ranked set sampling (RSS) is one of the most common and effective sampling designs, first proposed by [26]. Most statisticians favor using this sampling design since it provides more efficient estimates when compared to simple random sampling. McIntyre’s notion of ranking is feasible whenever it can be done easily by a judgment technique. For a detailed overview of the theory and applications of the RSS, see [27]. In some practical instances, the variable of main interest, say *Y*, is more difficult to measure than an auxiliary variable *X* related to *Y*, which is easily quantifiable and can be precisely arranged. In this instance, ref. [28] proposed an alternative RSS scheme, which is as follows:

1. Choose *m* independent bivariate samples, each of size *m*, at random.
2. Rank the units in each sample according to an auxiliary variable (X) together with its related variable (Y) .
3. Measure the $i^{th}$ observation of the $i^{th}$ set $\left(X_{{\left(i\right)}_{i}},Y_{{\left[i\right]}_{i}}\right)$ , i=1,⋯,m, where $X_{{\left(i\right)}_{i}}$ represents the $i^{th}$ OS of the $i^{th}$ sample and $Y_{{\left[i\right]}_{i}}$ represents the corresponding measurement conducted on the study variable *Y* of the same unit.
4. If a large sample size is required, repeat Steps 1 through 3 *d* times until a sample of size n=md is obtained, where *m* is the set size and *d* is the cycle count. Therefore, $\left(Y_{{\left[1\right]}_{1}},Y_{{\left[2\right]}_{2}},\ldots ,Y_{{\left[n\right]}_{n}}\right)$ constitutes a ranked set sample. Here, it is evident that $Y_{{\left[i\right]}_{i}}$ is the concomitant of $i^{th}$ OS arising from the $i^{th}$ sample, as coined by [29].

Ref. [30] provided a modified RSS method whereby only the greatest or smallest judgment-ranked unit is chosen for quantification. Suppose *n* random samples of size *n* are chosen from a bivariate distribution. From each of the *n* samples, select the unit with the largest (smallest) measurement on the auxiliary variable *X* and measure the *Y* variable related to it. The set of observations $Y_{{\left[n\right]}_{1}},Y_{{\left[n\right]}_{2}},\cdots ,Y_{{\left[n\right]}_{n}}$ $\left(Y_{{\left[1\right]}_{1}},Y_{{\left[1\right]}_{2}},\cdots ,Y_{{\left[1\right]}_{n}}\right)$ is called the upper RSS (URSS) (lower RSS (LRSS)). Multiple authors in the literature investigated the estimation of parameters for various bivariate distributions utilizing RSS and its modifications. In this field, some works were published by [23,24,31,32,33,34,35,36,37,38,39,40]. To the best of our knowledge, COSs from the BGLE distribution have not yet been studied. So, the objective of this study is to develop the distribution theory for COSs originating from the BGLE distribution and apply it to related inference issues.

The organization of the present paper is as follows: In Section 2, we present a general overview of the BGLE distribution and its characteristics, followed by a brief description of COSs. Section 3 provides the marginal PDF as well as the explicit formulas for the single moments of COSs from the BGLE distribution. Section 3 additionally presents the joint PDF of COSs from the BGLE distribution. Moreover, the explicit expressions for the product moments of COSs are derived. The best linear unbiased (BLU) estimator of a scale parameter related to a study variable, based on different RSS techniques, is obtained in Section 4. Then, in Section 5, we apply the paper’s results to a real dataset. Finally, Section 6 contains a conclusion.

## 2. Preliminaries

### 2.1. BGLE Distribution

The PDF of the BGLE distribution for a bivariate random variable (X,Y) with parameters $\left(\beta_{1},\beta_{2},\theta_{1},\theta_{2},\lambda \right)$ , as given by [41], is

$$\begin{array}{c} f\left(x,y\right)=\lambda \left(\beta_{1}+\theta_{1}x\right)\left(\beta_{2}+\theta_{2}y\right)e^{-\phi (x,y;\omega )}{\left(1-e^{-\phi (x,y;\omega )}\right)}^{\lambda -2}\left(1-\lambda e^{-\phi (x,y;\omega )}\right), \end{array}$$

where $x,y\geq 0,\;\beta_{i},\theta_{i}\geq 0$ such that $\beta_{i}+\theta_{i}>0$ for i=1,2 , and 0<λ≤1 , $\phi \left(x,y;\omega \right)=\beta_{1}x+\frac{\theta_{1}}{2}x^{2}+\beta_{2}y+\frac{\theta_{2}}{2}y^{2},$ and $\omega =(\beta_{1},\theta_{1},\beta_{2},\theta_{2})$ . Some specific distributions can be obtained from the BGLE distribution, as follows:

1. If $\theta_{1}=\theta_{2}=0,$ then the BGLE distribution becomes the BGE distribution. For details, see [10].
2. If $\beta_{1}=\beta_{2}=0,$ then the BGR distribution is obtained.

A series expansion of the PDF of the BGLE distribution (Pathak and Vellaisamy, [41]) is

f(x,y)=(β1+θ1x)(β2+θ2y)∑j=1∞λj(−1)j+1j2e−jϕ(x,y;ω).

The marginal PDFs of *X* and *Y* are given by ( Pathak and Vellaisamy [41])

$$\begin{array}{ccc} f_{X}\left(x\right) & = & \lambda \left(\beta_{1}+\theta_{1}x\right)e^{-(\beta_{1}x+\frac{\theta_{1}}{2}x^{2})}{\left(1-e^{-(\beta_{1}x+\frac{\theta_{1}}{2}x^{2})}\right)}^{\lambda -1},\;x\geq 0, \end{array}$$

and

$$\begin{array}{ccc} f_{Y}\left(y\right) & = & \lambda \left(\beta_{2}+\theta_{2}y\right)e^{-(\beta_{2}y+\frac{\theta_{2}}{2}y^{2})}{\left(1-e^{-(\beta_{2}y+\frac{\theta_{2}}{2}y^{2})}\right)}^{\lambda -1},\;y\geq 0, \end{array}$$

respectively. And the marginal CDFs of *X* and *Y* are as follows

$$\begin{array}{ccc} F_{X}\left(x\right) & = & {\left(1-e^{-(\beta_{1}x+\frac{\theta_{1}}{2}x^{2})}\right)}^{\lambda },\;x\geq 0, \end{array}$$

and

$$\begin{array}{ccc} F_{Y}\left(y\right) & = & {\left(1-e^{-(\beta_{2}y+\frac{\theta_{2}}{2}y^{2})}\right)}^{\lambda },\;y\geq 0, \end{array}$$

respectively. The conditional PDF of *Y* given X=x is given by (Pathak and Vellaisamy, [41])

$$\begin{array}{ccc} f(y|x) & = & \frac{\left(\beta_{2}+\theta_{2}y\right)e^{-(\beta_{2}y+\frac{\theta_{2}}{2}y^{2})}{\left(1-e^{-\phi (x,y;\omega )}\right)}^{\lambda -2}\left(1-\lambda e^{-\phi (x,y;\omega )}\right)}{{\left(1-e^{-(\beta_{1}x+\frac{\theta_{1}}{2}x^{2})}\right)}^{\lambda -1}}. \end{array}$$

Further, the bivariate ${\left(k_{1},k_{2}\right)}^{th}$ product moments of the BGLE distribution is given by (Pathak and Vellaisamy, [41])

(i) for $\beta_{1}=\beta_{2}=0$ and $\theta_{1},\theta_{2}>0$ , $k_{1},k_{2}=1,2,\ldots$ ,
(1) E(Xk1Yk2)=2(k1+k2)/2Γ(k1/2+1)Γ(k2/2+1)θ1k1/2θ2k2/2∑j=1∞λj(−1)j+11j(k1+k2)/2,
(ii) for $\beta_{1},\beta_{2}>0$ and $\theta_{1},\theta_{2}\geq 0$ , $k_{1},k_{2}=1,2,\ldots$ ,
(2) E(Xk1Yk2)=∑j=1∞∑t=0∞∑s=0∞λj(−1)j+t+s+1θ1tθ2sjt+s+22t+st!s!(β1j)k1+2t+1(β2j)k2+2s+1Γ(k1+2t+1)×Γ(k2+2s+1)(β1+θ1(k1+2t+1)β1j)(β2+θ2(k2+2s+1)β2j).

See [41] for a thorough proof of formulas (1) and (2), as well as more discussion.

Using the transformation $Z=\frac{X}{\theta_{1}^{⋆}}$ and $W=\frac{Y}{\theta_{2}^{⋆}}$ , $\theta_{i}^{⋆}=\theta_{i}^{-1/2},i=1,2$ , the standard BGLE distribution has the joint PDF as

(3) f⋆(z,w)=(β1θ1⋆+z)(β2θ2⋆+w)∑j=1∞λj(−1)j+1j2e−jϕ(z,w;ω),

where $\phi \left(z,w;\omega \right)=\left(\beta_{1}\theta_{1}^{⋆}z+\frac{z^{2}}{2}\right)+\left(\beta_{2}\theta_{2}^{⋆}w+\frac{w^{2}}{2}\right),$ and $\omega =(\beta_{1},\theta_{1}^{⋆},\beta_{2},\theta_{2}^{⋆})$ . Obviously, the variables *Z* and *W* follow the standard GLE distribution, and their corresponding PDFs are given as

(4) $$\begin{array}{ccc} f_{Z}^{⋆}\left(z\right) & = & \lambda \left(\beta_{1}\theta_{1}^{⋆}+z\right)e^{-(\beta_{1}\theta_{1}^{⋆}z+\frac{z^{2}}{2})}{\left(1-e^{-(\beta_{1}\theta_{1}^{⋆}z+\frac{z^{2}}{2})}\right)}^{\lambda -1},z\geq 0, \end{array}$$

(5) $$\begin{array}{ccc} f_{W}^{⋆}\left(w\right) & = & \lambda \left(\beta_{2}\theta_{2}^{⋆}+w\right)e^{-(\beta_{2}\theta_{2}^{⋆}w+\frac{w^{2}}{2})}{\left(1-e^{-(\beta_{2}\theta_{2}^{⋆}w+\frac{w^{2}}{2})}\right)}^{\lambda -1},w\geq 0. \end{array}$$

### 2.2. Concomitants of Order Statistics

Suppose $(X_{i},Y_{i}),\;i=1,2,\cdots ,n,$ is a random sample from a bivariate distribution with PDF f(x,y) . If we denote $X_{i:n}$ as the $i^{th}$ OS of the *X* sample values, then *Y* values associated with $X_{i:n}$ are called the concomitants of the $i^{th}$ OS and are symbolized by $Y_{\left[i:n\right]},i=1,2,\cdots ,n$ . For a detailed overview of concomitants of order statistics (COSs), we refer to [15,29].

The PDF of the concomitant of the $i^{th}$ OS is given by

(6) $$\begin{array}{c} h_{\left[i:n\right]}\left(y\right)=\int_{-\infty }^{\infty }f\left(y|x\right)f_{i:n}\left(x\right)dx, \end{array}$$

where f(y|x) is the conditional PDF of *Y* given X=x , and $f_{i:n}\left(x\right)$ is the PDF of the $i^{th}$ OS, which is given by

(7) $$\begin{array}{c} f_{i:n}\left(x\right)=C_{i:n}f\left(x\right){\left[F(x)\right]}^{i-1}{\left[1-F(x)\right]}^{n-i}, \end{array}$$

where $C_{i:n}=\frac{n!}{(i-1)!(n-i)!}$ . The relationship between the PDF of the concomitant of the first OS and the concomitant of the $i^{th}$ OS is given by (Balasubramanian and Beg [42])

(8) h[i:n](y)=∑s=n−i+1n(−1)s−n+i−1s−1n−insh[1:s](y).

Further, for 1≤i<j≤n, the joint PDF of concomitants of the $i^{th}$ and $j^{th}$ OSs is given by

(9) $$\begin{array}{c} h_{\left[i,j:n\right]}\left(y_{1},y_{2}\right)=\int_{-\infty }^{\infty }\int_{-\infty }^{x_{2}}f\left(y_{1}|x_{1}\right)f\left(y_{2}|x_{2}\right)f_{i,j:n}\left(x_{1},x_{2}\right)dx_{1}dx_{2}, \end{array}$$

where $f_{i,j:n}\left(x_{1},x_{2}\right)$ is the joint PDF of the $i^{th}$ and $j^{th}$ OSs and is given by

(10) $$\begin{array}{c} f_{i,j:n}\left(x_{1},x_{2}\right)=C_{i,j:n}f\left(x_{1}\right)f\left(x_{2}\right){\left[F(x_{1})\right]}^{i-1}{\left[F\left(x_{2}\right)-F\left(x_{1}\right)\right]}^{j-i-1}{\left[1-F(x_{2})\right]}^{n-j}, \end{array}$$

where $C_{i,j:n}=\frac{n!}{(i-1)!(j-i-1)!(n-j)!}$ (see [43,44]).

## 3. Distribution Theory of COSs from the BGLE Distribution

This section presents the distributions and moments of COSs arising from the BGLE distribution. Assume $\left(X_{i},Y_{i}\right)$ and $\left(Z_{i},W_{i}\right)$ are random samples of size *n* drawn from the BGLE distribution and the standard BGLE distribution, with PDFs given by (1) and (3), respectively. Let $W_{\left[r:n\right]}$ represent the concomitant of the $r^{th}$ OS $Z_{r:n}$ . The following theorem provides the PDF of $W_{\left[r:n\right]},$ where r=1,⋯,n .

**Theorem 1.** 
*If $W_{\left[r:n\right]}$ is the concomitant of the $r^{th}$ OS from the standard BGLE distribution, then the PDF $h_{\left[r:n\right]}\left(w\right)$ of $W_{\left[r:n\right]}$ , for 1≤r≤n, is given by*

(11) h[r:n](w)=∑s=n−r+1n(−1)s−n+r−1s−1n−rnsh[1:s](w),w≥0,

*where*

h[1:s](w)=s!(s−1)!∑i=0s−1∑j=1∞(−1)i+j+1s−1iλjj2(β2θ2⋆+w)

$$\times e^{-j(\beta_{2}\theta_{2}^{⋆}w+\frac{w^{2}}{2})}B\left(j,i\;\lambda +1\right),$$

*and B(.,.) is the complete beta function.*

**Proof.**  The PDF of the concomitant of the first OS $W_{\left[1:n\right]}$ is given by

(12) $$\begin{array}{c} h_{\left[1:n\right]}\left(w\right)=\int_{0}^{\infty }f\left(w|z\right)f_{1:n}\left(z\right)dz. \end{array}$$

In view of (3), (4), and (7), we get

(13) h[1:n](w)=n!(n−1)!∑i=0n−1∑j=1∞(−1)i+j+1n−1iλjj2(β2θ2⋆+w)×e−j(β2θ2⋆w+w22)I,

where

(14) $$\begin{array}{ccc} I & = & \int_{0}^{\infty }\left(\beta_{1}\theta_{1}^{⋆}+z\right)e^{-j(\beta_{1}\theta_{1}^{⋆}z+\frac{z^{2}}{2})}{\left(1-e^{-(\beta_{1}\theta_{1}^{⋆}z+\frac{z^{2}}{2})}\right)}^{i\;\lambda }dz, \end{array}$$

setting $t=e^{-(\beta_{1}\theta_{1}^{⋆}z+\frac{z^{2}}{2})}$ and $dt=-\left(\beta_{1}\theta_{1}^{⋆}+z\right)e^{-(\beta_{1}\theta_{1}^{⋆}z+\frac{z^{2}}{2})}dz$ in (14), we obtain

(15) $$\begin{array}{ccc} I & = & \int_{0}^{1}t^{j-1}{\left(1-t\right)}^{i\;\lambda }dt,\\ & & =B(j,i\;\lambda +1). \end{array}$$

Now, using (15) in (13), we get

(16) h[1:n](w)=n!(n−1)!∑i=0n−1∑j=1∞(−1)i+j+1n−1iλjj2(β2θ2⋆+w)×e−j(β2θ2⋆w+w22)B(j,iλ+1),w≥0.

Now, by using the relation (8), we obtain the result given in (11). □

The $k^{th}$ moments of $W_{\left[r:n\right]},$ 1≤r≤n , is given by the following theorem:

**Theorem 2.** 
*The $k^{th}$ moment of the concomitant of OS $W_{\left[r:n\right]},$ for k=1,2,⋯, is given by*

(17) μ[r:n](k)=∑s=n−r+1n(−1)s−n+r−1s−1n−rnsμ[1:s](k),

*where $\mu_{\left[1:s\right]}^{\left(k\right)}$ is given by*

*For $\beta_{2}=0,\;\theta_{2}^{⋆}>0,$
*

(18) μ[1:s](k)=s!(s−1)!∑i=0s−1∑j=1∞(−1)i+j+1s−1iλjB(j,iλ+1)2k/2Γ(k/2+1)jk/2−1,

*and for $\beta_{2}>0,\;\theta_{2}^{⋆}\geq 0,$
*

(19) μ[1:s](k)=s!(s−1)!∑i=0s−1∑j=1∞∑c=0∞(−1)i+j+c+1s−1iλjB(j,iλ+1)c!2c×Γ(k+2c+1)jk+c−1(β2θ2⋆)k+2c+1β2θ2⋆+k+2c+1jβ2θ2⋆.

**Proof.**  The $k^{th}$ moment of $W_{\left[r:n\right]}$ is given as

(20) $$\begin{array}{ccc} \mu_{\left[r:n\right]}^{\left(k\right)} & = & E\left[W_{\left[r:n\right]}^{k}\right]=\int_{0}^{\infty }w^{k}h_{\left[r:n\right]}\left(w\right)dw. \end{array}$$

Now, using (11), we get

(21) μ[r:n](k)=∑s=n−r+1n(−1)s−n+r−1s−1n−rnsμ[1:s](k),

where $\mu_{\left[1:s\right]}^{\left(k\right)}$ is the $k^{th}$ moment of $W_{\left[1:s\right]}$ , which is given as follows

(22) μ[1:s](k)=E[W[1:s]k]=∫0∞wkh[1:s](w)dw=s!(s−1)!∑i=0s−1∑j=1∞(−1)i+j+1s−1iλjj2B(j,iλ+1)I,

where

(23) $$\begin{array}{c} I=\int_{0}^{\infty }w^{k}\left(\beta_{2}\theta_{2}^{⋆}+w\right)e^{-j\beta_{2}\theta_{2}^{⋆}w}e^{-j\frac{w^{2}}{2}}dw. \end{array}$$

(1) When $\beta_{2}=0,\;\theta_{2}^{⋆}>0,$ we obtain
(24) $$\begin{array}{ccc} I & = & \int_{0}^{\infty }w^{k+1}e^{-j\frac{w^{2}}{2}}dw\\ & & =\frac{2^{k/2}\Gamma \left(k/2+1\right)}{j^{k/2+1}}. \end{array}$$

Using (24) in (22), we get (18).

(2) When $\beta_{2}>0,\;\theta_{2}^{⋆}\geq 0,$ using the following expansion:
$$e^{-j\frac{w^{2}}{2}}=\sum_{c=0}^{\infty }\frac{{\left(-1\right)}^{c}j^{c}w^{2c}}{c!\;2^{c}},$$

from (23), we have

(25) $$\begin{array}{ccc} I & = & \sum_{c=0}^{\infty }\frac{{\left(-1\right)}^{c}j^{c}}{c!\;2^{c}}\int_{0}^{\infty }\left(\beta_{2}\theta_{2}^{⋆}w^{k+2c}+w^{k+2c+1}\right)e^{-j\beta_{2}\theta_{2}^{⋆}w}dw\\ & & =\sum_{c=0}^{\infty }\frac{{\left(-1\right)}^{c}j^{c}}{c!\;2^{c}}\frac{\Gamma (k+2c+1)}{{\left(j\beta_{2}\theta_{2}^{⋆}\right)}^{k+2c+1}}\left[\beta_{2}\theta_{2}^{⋆}+\frac{k+2c+1}{j\beta_{2}\theta_{2}^{⋆}}\right]. \end{array}$$

Using (25) in (22), we get (19). □

**Theorem 3.** 
*The joint PDF of concomitants $W_{\left[r:n\right]}$ and $W_{\left[s:n\right]}$ , 1≤r<s≤n , is given by*

(26) $$\begin{array}{ccc} h_{\left[r,s:n\right]}\left(w_{1},w_{2}\right) & = & C_{r,s:n}\sum_{\ell_{1}=0}^{s-r-1}\sum_{\ell_{2}=0}^{n-s}\sum_{i_{1}=1}^{\infty }\sum_{i_{2}=1}^{\infty }\;\delta_{\lambda }\left(\ell_{1},\ell_{2},i_{1},i_{2}\right)\left(\beta_{2}\theta_{2}^{⋆}+w_{1}\right)\\ & & \times \left(\beta_{2}\theta_{2}^{⋆}+w_{2}\right)e^{-i_{2}\left(\beta_{2}\theta_{2}^{⋆}w_{1}+\frac{w_{1}^{2}}{2}\right)}e^{-i_{1}\left(\beta_{2}\theta_{2}^{⋆}w_{2}+\frac{w_{2}^{2}}{2}\right)},w_{1},\;w_{2}>0, \end{array}$$

*where*

$$\;\;\delta_{\lambda }\left(\ell_{1},\ell_{2},i_{1},i_{2}\right)=\phi_{\lambda }\left(\ell_{1},\ell_{2},i_{1},i_{2}\right)B\left(i_{1}+i_{2},\lambda \left(r+\ell_{1}-1\right)+1\right)$$

$$\times_{3}F_{2}\left(i_{1},-\lambda \left(s-r-\ell_{1}+\ell_{2}-1\right),i_{1}+i_{2};i_{1}+1,i_{1}+i_{2}+\lambda \left(r+\ell_{1}-1\right)+1;1\right),$$

ϕλ(ℓ1,ℓ2,i1,i2)=(−1)i1+i2+ℓ1+ℓ2+2i1i22s−r−1ℓ1n−sℓ2λi1λi2,

*where F23(a,b,c;d,e;x) denotes the hypergeometric function defined by*

F23(a,b,c;d,e;x)=∑j=0∞(a)j(b)j(c)j(d)j(e)jxjj!,

*and ${\left(k\right)}_{j}=k\left(k+1\right)\cdots \left(k+j-1\right)$ is the ascending factorial.*

**Proof.**  Using (9), the joint PDF of the $r^{th}$ and $s^{th}$ COS $W_{\left[r:n\right]}$ and $W_{\left[s:n\right]}$ is given as

(27) $$\begin{array}{c} h_{\left[r,s:n\right]}\left(w_{1},w_{2}\right)=\int_{0}^{\infty }\int_{z_{1}}^{\infty }f\left(w_{1}|z_{1}\right)f\left(w_{2}|z_{2}\right)f_{r,s:n}\left(z_{1},z_{2}\right)dz_{2}dz_{1} \end{array}$$

In view of (10), we get

(28) h[r,s:n](w1,w2)=Cr,s:n∑ℓ1=0s−r−1∑ℓ2=0n−s(−1)ℓ1+ℓ2s−r−1ℓ1n−sℓ2×∫0∞f(z1,w1)[F(z1)]r+ℓ1−1I(z1)dz1,

where

(29) I(z1)=∫z1∞f(z2,w2)[F(z2)]s−r−ℓ1+ℓ2−1dz2=∑i1=1∞λi1(−1)i1+1i12(β2θ2⋆+w2)e−i1(β2θ2⋆w2+w222)×∫z1∞(β1θ1⋆+z2)e−i1(β1θ1⋆z2+z222)(1−e−(β1θ1⋆z2+z222))dz2,

setting $t_{1}=e^{-(\beta_{1}\theta_{1}^{⋆}z_{2}+\frac{z_{2}^{2}}{2})}$ , we get

I(z1)=∑i1=1∞λi1(−1)i1+1i12(β2θ2⋆+w2)e−i1(β2θ2⋆w2+w222)×∫0ωt1i1−1(1−t1)λ(s−r−ℓ1+ℓ2−1)dt1,=∑i1=1∞λi1(−1)i1+1i12(β2θ2⋆+w2)e−i1(β2θ2⋆w2+w222)×Bω(i1,λ(s−r−ℓ1+ℓ2−1)+1),

where $\omega =e^{-(\beta_{1}\theta_{1}^{⋆}z_{1}+\frac{z_{1}^{2}}{2})}$ , and $B_{\omega }\left(a,b\right)$ is the incomplete beta function defined by $B_{\omega }\left(a,b\right)=\int_{0}^{\omega }x^{a-1}{\left(1-x\right)}^{b-1}dx.$

Placing the value of $I(z_{1})$ in (28), we obtain

(30) h[r,s:n](w1,w2)=Cr,s:n∑ℓ1=0s−r−1∑ℓ2=0n−s∑i1=1∞∑i2=1∞(−1)ℓ1+ℓ2+i1+i2+2s−r−1ℓ1n−sℓ2λi1×λi2i12i22(β2θ2⋆+w1)(β2θ2⋆+w2)e−i2(β2θ2⋆w1+w122)e−i1(β2θ2⋆w2+w222)×∫0∞(β1θ1⋆+z1)e−i2(β1θ1⋆z1+z122)(1−e−(β1θ1⋆z1+z122))λ(r+ℓ1−1)×Bω(i1,λ(s−r−ℓ1+ℓ2−1)+1)dz1.

Letting $t_{2}=e^{-(\beta_{1}\theta_{1}^{⋆}z_{1}+\frac{z_{1}^{2}}{2})}$ in (30), we get

(31) h[r,s:n](w1,w2)=Cr,s:n∑ℓ1=0s−r−1∑ℓ2=0n−s∑i1=1∞∑i2=1∞(−1)ℓ1+ℓ2+i1+i2+2s−r−1ℓ1n−sℓ2λi1×λi2i12i22(β2θ2⋆+w1)(β2θ2⋆+w2)e−i2(β2θ2⋆w1+w122)e−i1(β2θ2⋆w2+w222)×∫01t2i2−1(1−t2)λ(r+ℓ1−1)Bt2(i1,λ(s−r−ℓ1+ℓ2−1)+1)dt2.

We know that Bt(a,b)=taaF12(a,1−b;a+1;t) , and

(32) ∫01ta−1(1−t)b−1F12(c,d;e;t)dt=B(a,b)F23(c,d,a;e,a+b;1).

(see [45]). Now by using (32) in (31), we obtain the result of (26). □

**Theorem 4.** 
*The $k^{th}$ and $l^{th}$ moments of $W_{\left[r:n\right]}$ and $W_{\left[s:n\right]}$ is given as follows.*

*For $\beta_{2}=0,\;\theta_{2}^{⋆}>0,$
*

(33) $$\begin{array}{ccc} \mu_{\left[r,s:n\right]}^{\left(k,l\right)} & = & C_{r,s:n}\sum_{\ell_{1}=0}^{s-r-1}\sum_{\ell_{2}=0}^{n-s}\sum_{i_{1}=1}^{\infty }\sum_{i_{2}=1}^{\infty }\;\delta_{\lambda }\left(\ell_{1},\ell_{2},i_{1},i_{2}\right)\\ & & \times \frac{2^{\frac{k+l}{2}}}{i_{1}^{\frac{l}{2}+1}i_{2}^{\frac{k}{2}+1}}\Gamma \left(\frac{k}{2}+1\right)\Gamma \left(\frac{l}{2}+1\right), \end{array}$$

*and for $\beta_{2}>0,\;\theta_{2}^{⋆}\geq 0,$
*

(34) $$\begin{array}{ccc} \mu_{\left[r,s:n\right]}^{\left(k,l\right)} & = & C_{r,s:n}\sum_{\ell_{1}=0}^{s-r-1}\sum_{\ell_{2}=0}^{n-s}\sum_{i_{1}=1}^{\infty }\sum_{i_{2}=1}^{\infty }\sum_{c_{1}=0}^{\infty }\sum_{c_{2}=0}^{\infty }\frac{{\left(-1\right)}^{c_{1}+c_{2}}i_{1}^{c_{2}}i_{2}^{c_{1}}}{c_{1}!c_{2}!\;2^{c_{1}+c_{2}}}\;\delta_{\lambda }\left(\ell_{1},\ell_{2},i_{1},i_{2}\right)\\ & & \times \frac{\Gamma \left(k+2c_{1}+1\right)\Gamma \left(l+2c_{2}+1\right)i_{2}^{-(k+2c_{1}+1)}i_{1}^{-(l+2c_{2}+1)}}{\beta_{2}^{k+l+2c_{1}+2c_{2}+2}{\theta_{2}^{⋆}}^{k+l+2c_{1}+2c_{2}}}\\ & & \times \left(\beta_{2}+\frac{k+2c_{1}+1}{i_{2}\beta_{2}{\theta_{2}^{⋆}}^{2}}\right)\left(\beta_{2}+\frac{l+2c_{2}+1}{i_{1}\beta_{2}{\theta_{2}^{⋆}}^{2}}\right). \end{array}$$

*Here Γ(.) is the complete gamma function.*

**Proof.**  The $k^{th}$ and $l^{th}$ moments of $W_{\left[r:n\right]}$ and $W_{\left[s:n\right]}$ is given as

(35) $$\begin{array}{ccc} \mu_{\left[r,s:n\right]}^{\left(k,l\right)} & = & E\left[W_{\left[r:n\right]}^{k}W_{\left[s:n\right]}^{l}\right]=\int_{0}^{\infty }\int_{0}^{\infty }w_{1}^{k}w_{2}^{l}h_{\left[r,s:n\right]}\left(w_{1},w_{2}\right)dw_{1}dw_{2}\\ & & =C_{r,s:n}\sum_{\ell_{1}=0}^{s-r-1}\sum_{\ell_{2}=0}^{n-s}\sum_{i_{1}=1}^{\infty }\sum_{i_{2}=1}^{\infty }\;\delta_{\lambda }\left(\ell_{1},\ell_{2},i_{1},i_{2}\right)\;I, \end{array}$$

where

(36) $$\begin{array}{ccc} I & = & I_{1}I_{2}, \end{array}$$

where

$$I_{1}=\int_{0}^{\infty }w_{1}^{k}\left(\beta_{2}\theta_{2}^{⋆}+w_{1}\right)e^{-i_{2}\left(\beta_{2}\theta_{2}^{⋆}w_{1}+\frac{w_{1}^{2}}{2}\right)}dw_{1},$$

and

$$I_{2}=\int_{0}^{\infty }w_{2}^{l}\left(\beta_{2}\theta_{2}^{⋆}+w_{2}\right)e^{-i_{1}\left(\beta_{2}\theta_{2}^{⋆}w_{2}+\frac{w_{2}^{2}}{2}\right)}dw_{2}.$$

(1) When $\beta_{2}=0,\;\theta_{2}^{⋆}>0,$ we have
(37) $$\begin{array}{ccc} I & = & \int_{0}^{\infty }w_{1}^{k+1}e^{-i_{2}\frac{w_{1}^{2}}{2}}dw_{1}\int_{0}^{\infty }w_{2}^{l+1}e^{-i_{1}\frac{w_{2}^{2}}{2}}dw_{2}\\ & & =\frac{2^{\frac{k+l}{2}}}{i_{1}^{\frac{l}{2}+1}i_{2}^{\frac{k}{2}+1}}\Gamma \left(\frac{k}{2}+1\right)\Gamma \left(\frac{l}{2}+1\right). \end{array}$$

Using (37) in (35), we get (33).

(2) When $\beta_{2}>0,\;\theta_{2}^{⋆}\geq 0,$ using the following expansion:
$$e^{-i_{2}\frac{w_{1}^{2}}{2}}=\sum_{c_{1}=0}^{\infty }\frac{{\left(-1\right)}^{c_{1}}i_{2}^{c_{1}}w_{1}^{2c_{1}}}{c_{1}!\;2^{c_{1}}},$$

from (36), we have

(38) $$\begin{array}{ccc} I_{1} & = & \sum_{c_{1}=0}^{\infty }\frac{{\left(-1\right)}^{c_{1}}i_{2}^{c_{1}}}{c_{1}!\;2^{c_{1}}}\int_{0}^{\infty }w_{1}^{k+2c_{1}}\left(\beta_{2}\theta_{2}^{⋆}+w_{1}\right)e^{-i_{2}\beta_{2}\theta_{2}^{⋆}w_{1}}dw_{1}, \end{array}$$

(39) $$\begin{array}{ccc} I_{1} & = & \sum_{c_{1}=0}^{\infty }\frac{{\left(-1\right)}^{c_{1}}i_{2}^{c_{1}}}{c_{1}!\;2^{c_{1}}}\left[\beta_{2}\theta_{2}^{⋆}\int_{0}^{\infty }w_{1}^{k+2c_{1}}e^{-i_{2}\beta_{2}\theta_{2}^{⋆}w_{1}}dw_{1}+\int_{0}^{\infty }w_{1}^{k+2c_{1}+1}e^{-i_{2}\beta_{2}\theta_{2}^{⋆}w_{1}}dw_{1}\right]\\ & & =\sum_{c_{1}=0}^{\infty }\frac{{\left(-1\right)}^{c_{1}}i_{2}^{c_{1}}}{c_{1}!\;2^{c_{1}}}\frac{\Gamma (k+2c_{1}+1)}{{\left(i_{2}\beta_{2}\theta_{2}^{⋆}\right)}^{k+2c_{1}+1}}\left[\beta_{2}\theta_{2}^{⋆}+\frac{k+2c_{1}+1}{i_{2}\beta_{2}\theta_{2}^{⋆}}\right]. \end{array}$$

Similarly, using the expansion

$$e^{-i_{1}\frac{w_{2}^{2}}{2}}=\sum_{c_{2}=0}^{\infty }\frac{{\left(-1\right)}^{c_{2}}i_{1}^{c_{2}}w_{2}^{2c_{2}}}{c_{2}!\;2^{c_{2}}},$$

we have

(40) $$\begin{array}{ccc} I_{2} & = & \sum_{c_{2}=0}^{\infty }\frac{{\left(-1\right)}^{c_{2}}i_{1}^{c_{2}}}{c_{2}!\;2^{c_{2}}}\left[\beta_{2}\theta_{2}^{⋆}\int_{0}^{\infty }w_{2}^{l+2c_{2}}e^{-i_{1}\beta_{2}\theta_{2}^{⋆}w_{2}}dw_{2}+\int_{0}^{\infty }w_{2}^{l+2c_{2}+1}e^{-i_{1}\beta_{2}\theta_{2}^{⋆}w_{2}}dw_{2}\right]\\ & & =\sum_{c_{2}=0}^{\infty }\frac{{\left(-1\right)}^{c_{2}}i_{1}^{c_{2}}}{c_{2}!\;2^{c_{2}}}\frac{\Gamma (l+2c_{2}+1)}{{\left(i_{1}\beta_{2}\theta_{2}^{⋆}\right)}^{l+2c_{2}+1}}\left[\beta_{2}\theta_{2}^{⋆}+\frac{l+2c_{2}+1}{i_{1}\beta_{2}\theta_{2}^{⋆}}\right]. \end{array}$$

Using (39) and (40) in (35), we get (34).

□

Table 1 shows the means and variances of the COSs of the standard BGLE distribution for different choices of n,λ and $\beta_{2}$ . It is worth noting that the condition $\sum_{r=1}^{n}\mu_{\left[r:n\right]}^{j}=n\;\mu_{1:1}^{j},$ j=1,2 is fulfilled (see [29]). Table 1 displays that the variances are decreasing with respect to $\beta_{2}$ , while the means and variances are increasing with respect to λ .

From Theorem (2), the means and variances of the COSs $Y_{\left[r:n\right]}$ arising from the BGLE distribution are expressed as follows:

(41) $$\begin{array}{ccc} E[Y_{\left[r:n\right]}] & = & \theta_{2}^{⋆}E\left[W_{\left[r:n\right]}\right]\\ & & =\theta_{2}^{⋆}\mu_{\left[r:n\right]}, \end{array}$$

(42) $$\begin{array}{ccc} Var[Y_{\left[r:n\right]}] & = & {\theta_{2}^{⋆}}^{2}Var\left[W_{\left[r:n\right]}\right]\\ & & ={\theta_{2}^{⋆}}^{2}\delta_{r,r:n}, \end{array}$$

where $Var\left[W_{\left[r:n\right]}\right]=\mu_{\left[r:n\right]}^{\left(2\right)}-{\left(\mu_{\left[r:n\right]}\right)}^{2}$ , and r=1,⋯,n . The covariances between $Y_{\left[r:n\right]}$ and $Y_{\left[s:n\right]}$ are expressed using Theorems (2) and (4) as follows:

(43) $$\begin{array}{ccc} Cov[Y_{\left[r:n\right]},Y_{\left[s:n\right]}] & = & {\theta_{2}^{⋆}}^{2}Cov\left[W_{\left[r:n\right]},W_{\left[s:n\right]}\right]\\ & & ={\theta_{2}^{⋆}}^{2}\delta_{r,s:n}, \end{array}$$

where $Cov\left(W_{\left[r:n\right]},W_{\left[s:n\right]}\right)=\mu_{\left[r,s:n\right]}-\mu_{\left[r:n\right]}\mu_{\left[s:n\right]},$ and 1≤r<s≤n.

## 4. BLU Estimator of the Parameter $\theta_{2}^{⋆}$ Based on Different RSS Schemes

In this part, we obtain the best linear unbiased estimator of the parameter $\theta_{2}^{⋆}$ using RSS, LRSS, and URSS schemes, assuming the parameters $\beta_{2}$ and λ are known.

Suppose that the bivariate random vector (X,Y) follows the BGLE distribution with the PDF provided in (1). Choose a ranked set sample according to the Stokes RSS procedure. Let $X_{{\left(i:n\right)}_{i}}$ denote the observation obtained on the auxiliary variable *X* in the $i^{th}$ unit of the RSS, and let $Y_{{\left[i:n\right]}_{i}}$ denote the measurement made on the variable related with $X_{{\left(i:n\right)}_{i}},\;i=1,2,\cdots ,n.$ Clearly, $Y_{{\left[i:n\right]}_{i}}$ is the concomitant of the $i^{th}$ OS of a random sample of size *n* arising from BGLE distribution (refer to (p. 145, [29])). Let $Y_{\left[n\right]}=(Y_{{\left[1:n\right]}_{1}},Y_{{\left[2:n\right]}_{2}},$ ${\cdots ,Y_{{\left[n:n\right]}_{n}})}^{\prime }$ denote the column vector of the ranked set sample. According to (41) and (42), the mean and variance of $Y_{{\left[i:n\right]}_{i}},\;i=1,2,\cdots ,n$ are given below

(44) $$\begin{array}{c} E\left[Y_{{\left[i:n\right]}_{i}}\right]=\theta_{2}^{⋆}\mu_{\left[i:n\right]}, \end{array}$$

and

(45) $$\begin{array}{c} Var\left[Y_{{\left[i:n\right]}_{i}}\right]={\theta_{2}^{⋆}}^{2}\delta_{i,i:n}. \end{array}$$

Because $Y_{{\left[i:n\right]}_{i}}$ and $Y_{{\left[j:n\right]}_{j}},$ for (i≠j) , represent measurements on *Y* made from units engaged in two independent samples, the covariance between $Y_{{\left[i:n\right]}_{i}}$ and $Y_{{\left[j:n\right]}_{j}}$ is zero.

Then, from (44) and (45), we can write

(46) $$\begin{array}{c} E\left[Y_{\left[n\right]}\right]=\theta_{2}^{⋆}\mu , \end{array}$$

and the dispersion matrix of $Y_{\left[n\right]}$ ,

(47) $$\begin{array}{c} D\left[Y_{\left[n\right]}\right]={\theta_{2}^{⋆}}^{2}\Delta , \end{array}$$

where $\mu ={\left(\mu_{\left[1:n\right]},\cdots ,\mu_{\left[n:n\right]}\right)}^{\prime }$ and $\Delta =diag(\delta_{1,1:n},\delta_{2,2:n},\cdots ,\delta_{n,n:n}).$

If the parameters $\beta_{2}$ and λ involved in μ and Δ are known, then proceeding as in [29] (p. 185), the BLU estimator $\hat{\theta_{2}^{⋆}}$ of $\theta_{2}^{⋆}$ is obtained as

(48) $$\begin{array}{ccc} \hat{\theta_{2}^{⋆}} & = & {\left(\mu^{\prime }\Delta^{-1}\mu \right)}^{-1}\mu^{\prime }\Delta^{-1}Y_{\left[n\right]}\\ & & =\sum_{i=1}^{n}a_{i}Y_{{\left[i:n\right]}_{i}}, \end{array}$$

where $a_{i}=\frac{\mu_{\left[i:n\right]}/\delta_{i,i:n}}{\sum_{i=1}^{n}\mu_{\left[i:n\right]}^{2}/\delta_{i,i:n}}$ , and the variance of $\hat{\theta_{2}^{⋆}}$ is given by

(49) $$\begin{array}{ccc} Var[\hat{\theta_{2}^{⋆}}] & = & {\left(\mu^{\prime }\delta^{-1}\mu \right)}^{-1}{\theta_{2}^{⋆}}^{2}\\ & & ={\left(\sum_{i=1}^{n}\mu_{\left[i:n\right]}^{2}/\delta_{i,i:n}\right)}^{-1}{\theta_{2}^{⋆}}^{2}. \end{array}$$

Table 2 and Table 3 display the coefficients for the BLU estimator $\hat{\theta_{2}^{⋆}}$ of $\theta_{2}^{⋆}$ and $Var\left[\hat{\theta_{2}^{⋆}}\right]/{\theta_{2}^{⋆}}^{2}$ for various values of n,λ and $\beta_{2}$ .

We now present the BLU estimator of $\theta_{2}^{⋆}$ based on the URSS and LRSS. Let *n* random samples of size *n* be taken from the BGLE distribution. Choose the unit with the smallest (largest) measurement on the auxiliary variable *X* from each of the *n* samples. Then measure the *Y* variable related to it. The set of observations $Y_{{\left[1:n\right]}_{1}},Y_{{\left[1:n\right]}_{2}},\ldots ,Y_{{\left[1:n\right]}_{n}}$ ( $Y_{{\left[n:n\right]}_{1}},Y_{{\left[n:n\right]}_{2}},\ldots ,Y_{{\left[n:n\right]}_{n}}$ ) is referred to as the Lower RSS (LRSS) (Upper RSS (URSS)).

The BLU estimators ${\tilde{\theta }}_{2,LRSS}^{⋆}$ and ${\tilde{\theta }}_{2,URSS}^{⋆}$ of $\theta_{2}^{⋆}$ based on LRSS and URSS are

(50) $$\begin{array}{ccc} {\tilde{\theta }}_{2,LRSS}^{⋆} & = & \frac{1}{n\mu_{\left[1:n\right]}}\sum_{i=1}^{n}Y_{{\left[1:n\right]}_{i}}, \end{array}$$

(51) $$\begin{array}{ccc} {\tilde{\theta }}_{2,URSS}^{⋆} & = & \frac{1}{n\mu_{\left[n:n\right]}}\sum_{i=1}^{n}Y_{{\left[n:n\right]}_{i}}, \end{array}$$

and their variances are

(52) $$\begin{array}{ccc} Var[{\tilde{\theta }}_{2,LRSS}^{⋆}] & = & {\left(n\mu_{\left[1:n\right]}^{2}/\delta_{1,1:n}\right)}^{-1}{\theta_{2}^{⋆}}^{2}, \end{array}$$

(53) $$\begin{array}{ccc} Var[{\tilde{\theta }}_{2,URSS}^{⋆}] & = & {\left(n\mu_{\left[n:n\right]}^{2}/\delta_{n,n:n}\right)}^{-1}{\theta_{2}^{⋆}}^{2}. \end{array}$$

The efficiencies $e_{1}$ of ${\tilde{\theta }}_{2,LRSS}^{⋆}$ and $e_{2}$ of ${\tilde{\theta }}_{2,URSS}^{⋆}$ relative to $\hat{\theta_{2}^{⋆}}$ are given by

$$e_{1}=\frac{Var[\hat{\theta_{2}^{⋆}}]}{Var[{\tilde{\theta }}_{2,LRSS}^{⋆}]},\;\;\;\;e_{2}=\frac{Var[\hat{\theta_{2}^{⋆}}]}{Var[{\tilde{\theta }}_{2,URSS}^{⋆}]},$$

see, for instance, Refs. [24,46]. Table 4 displays the efficiencies $e_{1}$ and $e_{2}$ for n=2,⋯,5 , $\beta_{2}=0,0.5$ , and λ=0.50,0.90 . Based on Table 4, it is evident that:

1. The efficiency $e_{1}$ is less than one for all chosen values of $\beta_{2},\lambda$ , and *n*. Therefore, $\hat{\theta_{2}^{⋆}}$ is relatively more efficient than ${\tilde{\theta }}_{2,LRSS}^{⋆}$ . For a fixed pair $\left(n,\beta_{2}\right)$ , the efficiency $e_{1}$ increases as λ increases.
2. For all selected values of $\beta_{2},\lambda$ , and *n*, the efficiency $e_{2}$ is greater than one. Thereby, ${\tilde{\theta }}_{2,URSS}^{⋆}$ is relatively more efficient than $\hat{\theta_{2}^{⋆}}$ . For a fixed pair $\left(n,\beta_{2}\right)$ , the efficiency $e_{2}$ decreases with increasing λ .

## 5. Real Data Application

In this part, we present real data analysis to illustrate the utility of our procedure. We consider the real data set used by [41], originally taken from [47]. The data set in Table 5 represents the time (in minutes) of the first kick goal scored by any team (X) and the time of the first goal of any type scored by the home team (Y) . According to [41], the BGLE model is an appropriate fit for this data set. The estimators of $\beta_{1}$ , $\beta_{2}$ , $\theta_{1}$ , $\theta_{2}$ , and λ are, respectively, 0.00001, 0.00311, 0.00079, 0.00092, and 0.75905 (for additional details, see [41]). Using the data from Table 5, we generate random samples of size five. For RSS, we choose $m^{2}$ bivariate (X,Y) pairs, divide them into *m* sets of size *m*, and rank each set according to *X*. The $i^{th}$ order statistic of *Y* is chosen from the $i^{th}$ set ( i=1:m ), resulting in an RSS sample of size *m* every cycle. For URSS (LRSS), we select $m^{2}$ bivariate (X,Y) pairs, divide them into *m* sets of size *m*, and rank each set based on *X*. We select the unit with the greatest (smallest) measurement on the variable *X* and measure the *Y* variable associated with it, resulting in a URSS (LRSS) sample of size *m* per cycle. This technique is repeated *d* times to obtain (n=m×d) . Here, we take m=5, and d=1 . Table 6 displays the samples under RSS schemes.

The proposed estimator of $\theta_{2}^{⋆}$ under various RSS schemes depends on the parameters $\beta_{2}$ and λ , which are unknown in this case. Thus, the estimators of these parameters can be obtained using the moment estimation approach (see [38], for example). Here, assuming that $\beta_{2}=0$ , we use a moment equation based on the correlation between $(X_{{\left(i:n\right)}_{i}},Y_{{\left[i:n\right]}_{i}}),i=1,2,\cdots ,n,$ to get the moment estimator of λ . This yields $\hat{\lambda }=0.40844$ . Table 7 displays the estimates of $\theta_{2}^{⋆}$ using the RSS, LRSS, and URSS techniques. The findings indicate that ${\tilde{\theta }}_{2,URSS}^{⋆}$ has the smallest variance. This is in line with the efficiency performance study’s results, which are shown in Section 4.

## 6. Conclusions

The BGLE distribution was presented by [41] as a new flexible bivariate distribution. This study examined the COSs from this distribution and derived explicit expressions for single and product moments of COSs. The BLU estimator of the scale parameter associated with the study variable is obtained under different RSS techniques. Additionally, a real data example is provided. The numerical findings emphasize that the BLU estimate under the URSS scheme is more efficient than the BLU estimates under the RSS and LRSS schemes.

## Figures and Tables

**Table 1:** Mean and variance of the COS for the standard BGLE distribution for different choices of n,λ and $\beta_{2}$ .

			Mean		Variance	
*λ*	*n*	*r*	*β*_2_ = 0	*β*_2_ = 0.5	*β*_2_ = 0	*β*_2_ = 0.5
0.50	1	1	0.88537	0.03669	0.44316	0.00253
	2	1	0.71254	0.02719	0.40123	0.00195
		2	1.05819	0.04620	0.42535	0.00293
	3	1	0.60479	0.02178	0.36218	0.00158
		2	0.92804	0.03801	0.40967	0.00249
		3	1.12327	0.05030	0.42049	0.00309
	4	1	0.52898	0.01823	0.32945	0.00134
		2	0.83224	0.03242	0.39139	0.00217
		3	1.02385	0.04360	0.40958	0.00275
		4	1.15641	0.05253	0.41974	0.00319
	5	1	0.47183	0.01570	0.30217	0.00116
		2	0.75755	0.02833	0.37327	0.00193
		3	0.94427	0.03856	0.39765	0.00248
		4	1.07690	0.04695	0.41050	0.00290
		5	1.17629	0.05393	0.42007	0.00325
0.90	1	1	1.19665	0.05581	0.43482	0.00340
	2	1	1.16472	0.05370	0.43955	0.00332
		2	1.22858	0.05792	0.42805	0.00346
	3	1	1.14197	0.05225	0.44355	0.00328
		2	1.21022	0.05659	0.42845	0.00341
		3	1.23776	0.05858	0.42760	0.00349
	4	1	1.12415	0.05115	0.44699	0.00324
		2	1.19543	0.05556	0.42942	0.00337
		3	1.22500	0.05763	0.42705	0.00345
		4	1.24201	0.05890	0.42771	0.00350
	5	1	1.10944	0.05025	0.45000	0.00321
		2	1.18299	0.05471	0.43061	0.00334
		3	1.21411	0.05683	0.42704	0.00341
		4	1.23226	0.05816	0.42692	0.00347
		5	1.24445	0.05909	0.42788	0.00351

**Table 2:** The coefficients for the BLU estimator $\hat{\theta_{2}^{⋆}}$ of $\theta_{2}^{⋆}$ and $Var\left[\hat{\theta_{2}^{⋆}}\right]/{\theta_{2}^{⋆}}^{2}$ for λ=0.50 .

*β*_2_	*n*	Coefficients					$\mathrm{Var}\left[\hat{\theta_{2}^{⋆}}\right]/{\theta_{2}^{⋆}}^{2}$
		*a*_1_	*a*_2_	*a*_3_	*a*_4_	*a*_5_	
0	1	1.12947					0.56534
	2	0.45559	0.63823				0.25654
	3	0.27317	0.37059	0.43700			0.16359
	4	0.19196	0.25422	0.29886	0.32939		0.11956
	5	0.14682	0.19082	0.22327	0.24667	0.26329	0.09402
0.5	1	27.2517					1.87608
	2	12.5946	14.2327				0.90133
	3	8.09753	8.98734	9.58394			0.58924
	4	5.94968	6.51752	6.92761	7.19962		0.43679
	5	4.69772	5.09538	5.39554	5.61122	5.75510	0.34671

**Table 3:** The coefficients for the BLU estimator $\hat{\theta_{2}^{⋆}}$ of $\theta_{2}^{⋆}$ and $Var\left[\hat{\theta_{2}^{⋆}}\right]/{\theta_{2}^{⋆}}^{2}$ for λ=0.90 .

*β*_2_	*n*	Coefficients					$\mathrm{Var}\left[\hat{\theta_{2}^{⋆}}\right]/{\theta_{2}^{⋆}}^{2}$
		*a*_1_	*a*_2_	*a*_3_	*a*_4_	*a*_5_	
0	1	0.83567					0.30365
	2	0.40073	0.43405				0.15123
	3	0.25898	0.28413	0.29117			0.10059
	4	0.18944	0.20970	0.21607	0.21874		0.07533
	5	0.14840	0.16536	0.17113	0.17374	0.17507	0.06019
0.5	1	17.9185					1.09091
	2	8.79811	9.10867				0.54458
	3	5.78630	6.02338	6.09041			0.36272
	4	4.29253	4.48448	4.54722	4.57140		0.27187
	5	3.40265	3.56384	3.62140	3.64662	3.65790	0.21740

**Table 4:** Efficiencies of the estimators ${\tilde{\theta }}_{2,LRSS}^{⋆}$ and ${\tilde{\theta }}_{2,URSS}^{⋆}$ relative to $\hat{\theta_{2}^{⋆}}$ .

		$e_{1}$		$e_{2}$	
*n*	*λ*	*β*_2_ = 0	*β*_2_ = 0.5	*β*_2_ = 0	*β*_2_ = 0.5
2	0.50	0.64926	0.68484	1.35074	1.31516
3	0.50	0.49563	0.52902	1.47260	1.44616
4	0.50	0.40617	0.43380	1.52362	1.51285
5	0.50	0.34637	0.36881	1.54852	1.55178
2	0.90	0.93347	0.94487	1.06654	1.05513
3	0.90	0.88724	0.90699	1.08120	1.07036
4	0.90	0.85183	0.87817	1.08669	1.07702
5	0.90	0.82321	0.85497	1.08932	1.08065

**Table 5:** Data for the UEFA Champions League from 2004 to 2006.

*X*	*Y*	*X*	*Y*	*X*	*Y*	*X*	*Y*
26	20	82	48	34	34	25	14
63	18	72	72	53	39	55	11
19	19	66	62	54	7	49	44
66	85	25	9	51	28	24	24
40	40	41	3	76	64	44	30
49	49	16	75	64	15	27	27
8	8	18	18	26	48	28	28
69	71	22	14	16	16	2	2
39	39	36	52	44	13		

**Table 6:** Samples of size n=5 under different RSS techniques.

Technique	*n*	Sample Values for Y-Variable				
RSS	5	16	3	42	62	18
LRSS	5	16	3	8	24	3
URSS	5	72	85	48	64	18

**Table 7:** The estimates of $\theta_{2}^{⋆}$ under different RSS techniques.

Technique	*n*	Estimator of $\theta_{2}^{⋆}$	Estimate of $\theta_{2}^{⋆}$	$\mathrm{Var}\left[\hat{\theta_{2}^{⋆}}\right]/{\theta_{2}^{⋆}}^{2}$	$\mathrm{SE}(\hat{\theta_{2}^{⋆}})$
RSS	5	$\hat{\theta_{2}^{⋆}}$	35.8391	0.10984	11.878
LRSS	5	${\tilde{\theta }}_{2,LRSS}^{⋆}$	33.5106	0.42238	21.779
URSS	5	${\tilde{\theta }}_{2,URSS}^{⋆}$	50.1818	0.06383	12.678

## Data Availability

Data are contained within the article.

## Abbreviations

The following abbreviations are used in this manuscript:

GLE	Generalized linear exponential
BGLE	Bivariate generalized linear exponential
BGE	Bivariate generalized exponential
BGR	Bivariate generalized Rayleigh
PDF	Probability density function
CDF	Cumulative distribution function
COSs	Concomitants of order statistics
RSS	Ranked set sampling
URSS	Upper ranked set sampling
LRSS	Lower ranked set sampling
BLU	Best linear unbiased
